# Juror Characteristics and Decision Making in a Developed Coercive Control Case

**DOI:** 10.3390/bs15060803

**Published:** 2025-06-12

**Authors:** Kacey May Barnett, Russell Woodfield, Rachel A. Conlon

**Affiliations:** School of Psychology, Leeds Trinity University, Leeds LS18 5HD, UK

**Keywords:** coercive control, jury decision making, psychopathic personality traits, dating violence attitudes

## Abstract

The aim of this study was to investigate whether juror characteristics, namely, age, attitudes surrounding coercive control and psychopathic personality traits (PPT), can influence Guilty or Not Guilty verdicts in a developed coercive control trial. One hundred and thirty-five participants (N = 135) completed an online survey consisting of elements of a mock coercive control trial and three questionnaires: the Coercive Control subsection of the Modern Adolescent Dating Violence Attitudes (MADVA (CC)) Scale, the Psychopathic Personality Traits Scale—Revised (PPTS-R) and the Juror Decision Scale (JDS). The results of the analysis demonstrated significant positive correlations between MADVA (CC) scores and all four subscales of the PPTS-R, highlighting the relationship between psychopathy traits and coercive control attitudes. Binary logistic regression findings showed that higher scores on the MADVA (CC) Scale were the only significant predictor of returning a Not Guilty verdict. Those who also returned a Not Guilty verdict had more accepting controlling behaviour attitudes, scored higher for defendant believability and were less confident in their overall decision. Findings from the current study highlight the significance of attitudes in a juror decision-making context. The significance of attitudes may also be applicable to police officers and other agencies within the criminal justice system. Additional efforts need to be made regarding the identification of coercive control tactics, and training programmes should be implemented within the police to increase identification of these behaviours in order and to improve case progression. This may increase the likelihood of a jury being required in these cases. Furthermore, Not Guilty verdicts were given with significantly less confidence than Guilty verdicts, although they have the same influence at trial. More research needs to be carried out to explore the development and maintenance of accepting attitudes towards coercive control, and there is a need for better education regarding coercive control to attempt to tackle harmful attitudes towards it and aim for fairer trials.

## 1. Introduction

Coercive control (CC) is a type of abuse involving one individual exerting power over another, resulting in a loss of dignity and respect and leaving victims fearful and trapped (Pitman, 2016; Stark, 2007, 2013). Tactics can include monitoring movements and restricting a person’s access to employment and medical services (Pitman, 2016; Stark, 2007, 2013). Coercive control became a criminal offence in the UK in 2015 under Section 76 of the Serious Crime Act. It is identified as ‘controlling or coercive behaviour in an intimate or family relationship’ (Serious Crime Act, 2015). If a person is convicted, the maximum sentence length is five years’ imprisonment (CPS, 2023b). In the year ending in March 2024, there were 851,062 domestic abuse-related crimes recorded by the police in England and Wales (ONS, 2024a). Over 45,000 of these reports were coercive control offences (ONS, 2024b). Less than 5% of all recorded domestic abuse-related incidents resulted in convictions (38,776 offenders convicted) (ONS, 2024c). Conviction rates can be explored through understanding juror decision making at trial.

The United Kingdom (UK) has adopted a jury system whereby lay individuals are involved in criminal proceedings to ensure a fair and just process where convictions are concerned (GOV.UK, 2018). On occasion, forensic experts may provide specialist knowledge to help jurors understand complex evidence and make informed decisions about a case (CPS, 2023a). Buongiorno et al. (2025) argue that forensic experts have their own interpretative biases; therefore, in a juror context, this may influence and persuade the jurors, creating additional complexities regarding decision making. Furthermore, irrespective of forensic experts, the juror members may still draw different conclusions regarding the evidence, and may therefore be unable to reach a unanimous decision regarding the defendants’ guilt (Singleton, 2024). This suggests that decision making is influenced by factors independent of the courtroom (Ellsworth, 1993); therefore, depending on the characteristics of the juror members, the process may not be fair and just. Mock jury research aims to simulate real-world criminal cases to investigate the factors that may influence verdict outcomes (Bornstein, 2017). Mock jury research has been criticised for using college and university samples (Nuñez et al., 2011), with Mills and Bohannon (1980) suggesting that age and education in college samples impact decision making. Therefore, community samples are necessary and allow researchers to assess whether there are any significant findings associated with age that may not be accessible within a student sample. Some mock jury research uses experimental designs and incorporates group deliberations (Lilley et al., 2023; Sommers, 2006; Willmott et al., 2018). However, other mock jury research solely focuses on individual juror processing (Gunnell & Ceci, 2010) and criticises jury deliberation for creating additional bias (Curley et al., 2022). Jury simulation research generally uses either written or video vignettes to encompass a criminal case (Bieneck, 2015). Vignettes are a good way to present relevant information to jurors and are a valuable method to gain knowledge relating to attitudes and beliefs about a subject (Bieneck, 2015).

Current and previous mock jury research is generally concerned with sexual violence trials (Curley et al., 2022; Helm & Growns, 2022) and rape cases (Ellison & Munro, 2009). This could be due to the lack of physical evidence often associated with these cases (Hritz et al., 2015; Menaker & Cramer, 2012), which is also similar in a coercive control trial. Pennington and Hastie (1992) proposed the ‘Story Model’ for cases where compelling versions of similar accounts are heard at trials lacking physical evidence. In such cases, jurors are required to choose which ‘story’ they believe, which can be quantitatively measured using the Juror Decision Scale (JDS; Willmott et al., 2018). This breaks down the overall verdict decision and considers complainant believability, defendant believability and overall confidence in decision making (Willmott et al., 2018). This is a more in-depth way of analysing verdict decisions and is especially useful when analysing individual decision making. Willmott et al. (2018) used the JDS alongside assessing attitudes in a mock rape trial and found that positive attitudes towards the complainant were associated with greater complainant believability and subsequent Guilty verdicts. Therefore, the likelihood of the juror member believing either the complainant or the defendant may be influenced by their preconceived ideas about a particular crime (Pennington & Hastie, 1992), as well as their own backgrounds and ideologies (Lilley et al., 2023).

Juror attitudes have resulted in bias in decision making and verdict outcomes (Curley et al., 2022). Previous mock jury research has examined the role of rape myth acceptance (RMA) in rape trials and found that higher endorsement of RMA can predict Not Guilty verdicts (Lilley et al., 2023; Willmott et al., 2018). Stevens et al. (2023) also found RMA to be a predictor of greater defendant believability in a domestic sex trafficking case. Therefore, research has shown that negative attitudes and beliefs can hinder the justice process (Lilley et al., 2023; Stevens et al., 2023; Willmott et al., 2018). There is limited research measuring attitudes towards coercive control specifically; however, there are similarities to attitudes that are accepting of rape myths as both attitudes aim to blame the victim and excuse the behaviour of the perpetrator (Lonsway & Fitzgerald, 1994; Stark, 2007). It seems necessary to assess attitudes surrounding coercive control amongst jurors as this could influence decision making, as previously seen with those who are accepting of rape myths (Lilley et al., 2023; Stevens et al., 2023; Willmott et al., 2018).

Research has found an association between attitudes and psychopathic personality traits (PPT) (Watts et al., 2017; Willmott et al., 2024), with higher levels of psychopathic traits being significantly associated with endorsement of rape myths. Psychopathic personality traits have been found to be associated with younger ages (Bate et al., 2014), suggesting that, as age increases, interpersonal and affective psychopathy traits decrease. Psychopathy is a personality construct characterised by a combination of affective (e.g., reduced guilt and empathy), interpersonal (e.g., interpersonal manipulation) and behavioural (e.g., impulsivity) facets (Gillespie et al., 2022; Hare, 1991; Patrick et al., 2009). Psychopathy was originally conceptualised to include a factor of criminal behaviour as a defining consequence of its presence in an individual (Hare, 1991). However, recent theory and research have suggested that PPT exist on a continuum in individuals and are not limited to forensic populations. As such, criminal behaviour may be an outcome behaviour of psychopathy and is not a defining factor in its presence, as highlighted in non-offending research populations (Boduszek et al., 2022). It is important to assess the psychopathic personality traits of juror members, as Durand et al. (2017) found that those who scored highly for PPT in a community sample were less likely to stigmatise an offender with psychopathy, downplay their violence and normalise aggressive behaviours. Therefore, had this offender been on trial for a violent offense and these community members serving on the jury, there may have been some bias, as higher levels of PPT in juror members could result in more Not Guilty verdicts. This is particularly relevant in a coercive control case as psychopathy is a possible characteristic present in CC perpetrators (Day & Bowen, 2015).

Enander (2010) found that domestic abuse perpetrators were described as being ‘sociable, charismatic and charming’ by their survivors, which could be interpreted as showing characteristics associated with psychopathy (Gillespie et al., 2022; Patrick et al., 2009). Furthermore, these traits were used manipulatively in an outward social context, while abusive, controlling behaviours were displayed privately (Enander, 2010). Describing the perpetrators as ‘psychopaths’ in this study had a legitimising effect, thus resulting in the victims justifying the abuse they endured (Enander, 2010). In a juror decision-making context, a defendant with a label of ‘psychopathy’ has the opposite effect, as this results in more Guilty verdicts being returned (Blais & Forth, 2013; Cox et al., 2016; Edens et al., 2003). However, findings from Durand et al. (2017) suggest that this may not always be the case; therefore, it is important to assess the PPT of the jurors themselves to see whether this has any influence on decision making. Gunschera et al. (2022) suggest that psychopathy is consistently linked with poor decision making; however, this is a rarely researched area with regard to juror decision making.

Psychopathic traits can be measured using the Psychopathic Personality Traits Scale—Revised (PPTS-R; Boduszek et al., 2022), which considers four factors: cognitive responsiveness, egocentricity, affective responsiveness and interpersonal manipulation. Cognitive responsiveness refers to the ability to understand others’ emotions and take another person’s perspective (Boduszek et al., 2017). Leith and Baumeister (1998) suggest that those who lack perspective-taking abilities are more likely to show rigidity in their thinking and aversion to compromise, and to be extremely judgemental. Furthermore, egocentricity is associated with low emotional empathy; those who are egocentric are only concerned with their own needs (Raine & Uh, 2018). While it is clear that these traits are problematic in a juror decision-making context, these traits alone may not be substantial enough to impact verdict outcomes. Considering the interplay of attitudes and psychopathic traits, as seen in the studies of Watts et al. (2017) and Willmott et al. (2024), it is suggested that these traits may result in bias when accompanied by more negative victim attitudes. Watts et al. (2017) found that a lack of empathy was associated with higher endorsement of RMA, misconceptions and stereotypes. Endorsement of rape myths can influence decision making (Lilley et al., 2023; Willmott et al., 2018); therefore, it is suggested that the combination of problematic attitudes and a lack of empathy may be more influential in the decision-making process than deficits in cognitive responsiveness or higher egocentricity alone. To the researcher’s knowledge, the relationship between PPT and attitudes towards coercive control is a new research area.

Overall, the literature presented suggests that attitudes and psychopathic traits can influence verdict outcomes in a variety of trials. Negative victim attitudes in sexual violence trials have been associated with higher levels of psychopathic traits (Watts et al., 2017; Willmott et al., 2024). Psychopathic trait levels are higher at younger ages (Bate et al., 2014) and may impact verdict outcomes if identification with the defendant occurs (Durand et al., 2017). Therefore, it seems necessary to assess psychopathic traits amongst jurors. Attitudes surrounding coercive control specifically is a limited research area, with its application to a juror context being seemingly non-existent. Therefore, there are clear gaps in the literature that need to be explored.

### The Current Study

The rationale for the forthcoming study considers all previous gaps in the literature, with the objective to examine a variety of psychological constructs with regard to individual decision making in an online developed coercive control trial. The current research seeks to test whether juror characteristics, such as age, attitudes towards coercive control and PPT, can influence decision making in a mock coercive control trial and the subsequent verdict outcomes.

The current study has a number of aims:

To explore the relationship between all independent variables: age, scores on the Coercive Control subsection of the Modern Adolescent Dating Violence Attitudes (MADVA (CC)) Scale and scores on the Psychopathic Personality Traits Scale—Revised (PPTS-R);

To investigate whether any of these independent variables could impact the likelihood of a Guilty or Not Guilty verdict being returned;

To assess whether there is a significant difference in scores for complainant and defendant believability on the Juror Decision Scale (JDS) and subsequent verdict decisions;

To investigate whether there is a significant difference in confidence levels between Guilty and Not Guilty verdicts by assessing overall decision confidence scores.

Based on the previous literature, it was hypothesised that younger ages would be associated with higher levels of psychopathic personality traits (Bate et al., 2014). It was anticipated that scores on the Coercive Control subscale of the MADVA Scale would be significantly positively correlated with scores on the PPTS-R, as this was found to be the case with RMA scores previously (Watts et al., 2017; Willmott et al., 2018). Previous research has found that both attitudes and psychopathic personality traits can influence decision making (Curley et al., 2022; Durand et al., 2017; Gunschera et al., 2022; Lilley et al., 2023; Stevens et al., 2023; Willmott et al., 2018). Therefore, it was hypothesised that both of these variables would be significant predictors of verdict outcomes, with higher scores on the Coercive Control subsection of the MADVA Scale and higher scores on the PPTS-R significantly returning more Guilty verdicts. Based on previous juror research, it was anticipated that those who returned a Guilty verdict would also score higher for complainant believability and be more confident in their decision (Willmott et al., 2018).

## 2. Method

### 2.1. Design

This was a quantitative study with a cross-sectional design. The study was conducted via JISC Online Surveys (Jisc, 2025) and participants were instructed to complete the survey once. The survey adopted elements of a developed coercive control trial. Participants (see Results section for details) from the general public in the United Kingdom were recruited using convenience sampling as well as using the researcher’s university research participation scheme for students and utilising social media platforms (Facebook, Instagram and LinkedIn).

### 2.2. Ethics and Procedure

The study was conducted in accordance with the Declaration of Helsinki and approved by the FSSE Ethics Committee, Leeds Trinity University (UK), on 24 May 2024, in line with the British Psychological Society Code of Human Research Ethics and Ethics and Conduct (BPS, 2021a, 2021b). All collected data was handled in line with the researcher’s university’s GDPR (General Data Protection Regulation) guidelines. The data was stored on a private, password-protected server at Leeds Trinity University and will remain on the server for 10 years, after which it will be disposed of safely and securely. All participants were made aware of this, and that all data collected would be recorded and stored anonymously. Inclusion criteria required participants to be UK residents as the study was based on laws from England and Wales. Therefore, the exclusion criteria excluded anyone not residing in the UK at the time of the study. Participants also had to be 18 years of age to participate as they were able to give informed consent without a parent or guardian, as per the British Psychological Society (BPS) guidelines (Oates et al., 2021).

The study was accessed by participants via JISC Online Surveys (Jisc, 2025). All sections of the study were mandatory, and progression could not occur unless all sections were answered. Participants were first provided with an information sheet outlining the details of the study and the contact details of the researchers. They were informed that they could withdraw at any time throughout the survey by simply closing their internet browser (incomplete data was excluded from the subsequent analysis). Participants were also informed that data could also be withdrawn after the study by contacting the researchers (no requests were made for withdrawal of data). Data collection occurred over a time period of approximately 4 weeks before the survey was closed. Participants were then given a standardised consent form to complete. Participants were asked about their demographic information, including age, gender identity and whether they were a UK resident.

They were then asked to complete the Coercive Control subsection of the MADVA Scale. This was followed by the PPTS-R. Following this, participants were shown elements of a hypothetical coercive control trial, including background information about the case, the allegation and legislation surrounding coercive control laws in England and Wales (see Appendix A). They were then shown juror instructions before being taken to the Prosecution case. After reading all of the information, participants were instructed to click ‘next’, whereafter they were shown the Defence case. Participants were then asked to continue to the following page, where they were given final instructions and asked to return their verdict decision. Participants then completed the JDS to assess their confidence in their verdict outcome. Finally, a debrief page was shown, careful consideration was given to ensure appropriate helplines were given (surrounding the potentially harmful nature of the research) and participants were provided with the contact details of the researchers in case they had any questions or wished to withdraw data at a later time point (up to 2 weeks after the close of the survey).

### 2.3. Materials

The Coercive Control subsection of the *Modern Adolescent Dating Violence Attitudes* (MADVA; Kirkman et al., 2025) Scale was used in the current study (items 41–48). The MADVA Scale is a scenario-based attitudinal measurement scale concerned with male-perpetrated violence against female victims. The statements each have a 5-point Likert scale (1 = *Strongly Disagree* to 5 = *Strongly Agree*), whereby participants decide how much they agree with each statement. In the current study, Cronbach’s Alpha was calculated as 0.85.

The *Psychopathic Personality Traits Scale—Revised* (PPTS-R; Boduszek et al., 2022) is a self-report 28-item measure designed to assess psychopathic traits in forensic and non-forensic populations. The PPTS-R consists of four subscales: affective responsiveness (AFFRES), cognitive responsiveness (COGRES), interpersonal manipulation (INTMAN) and egocentricity (EGO). All four subscales are made up of seven items scored on a 5-point Likert scale (1 = *Strongly Disagree* to 5 = *Strongly Agree*). Cronbach’s Alpha was calculated for each subscale, proving excellent internal reliability (AFFRES = 0.95, COGRES = 0.78, INTMAN = 0.91, EGO = 0.86) (Boduszek et al., 2022). In the current study, Cronbach’s Alpha was calculated for the four subscales as AFFRES = 0.78, COGRES = 0.63, INTMAN = 0.88 and EGO = 0.78.

### 2.4. Coercive Control Scenario Development

The mock trial materials were inspired by the work of Lilley et al. (2023). The Prosecution and Defence were written vignettes and were specifically developed scenarios for the purpose of this study (see Appendix A). This was achieved by using the Crown Prosecution Service (CPS) guidelines for a coercive control prosecution (CPS, 2023a) to ensure the study aimed for ecological validity. The scenarios also incorporated elements from the Coercive Control subsection of the MADVA Scale to ensure consistency and coherence between the scale and scenario and to make sure the internal validity of the study was maintained. Furthermore, Walker et al. (2024) used written vignettes to explore bystander intervention in a coercive control scenario; therefore, some elements from this vignette were adapted and utilised for the current study. The final scenarios were overlooked by an expert in the field.

*The Juror Decision Scale* (JDS; Willmott et al., 2018) is a 16-item self-report measure designed to assess individual juror decision making. The measure consists of three subscales: complainant believability, defendant believability and decision confidence. Both complainant believability and defendant believability include seven items, with decision confidence including two. All items are scored on a 5-point Likert scale (1 = “not at all” to 5 = “extremely”). Composite reliability was used in the original study to assess the internal reliability of the JDS factors, with values above 0.60 typically considered acceptable (Diamantopoulos & Siguaw, 2000; Willmott et al., 2018). In the current study, Cronbach’s Alpha was calculated for the three subscales as overall decision confidence = 0.72, defendant believability = 0.75 and complainant believability = 0.79.

### 2.5. Analysis

The current study utilised several statistical procedures for the analysis using SPSS, 29 (IBM Corp, 2023) and R Statistical Software (v4.5.0; R Core Team, 2025). Pearson’s product–moment correlation coefficient was used to ascertain the strength of the relationship between variables as it is the recommended standard analysis method (Tabachnick & Fidell, 2013). Binary logistic regression was used to ascertain if any independent variables (IV) could predict the dependent variable (DV) with only two outcomes (Miles & Shevlin, 2001). T-tests were used to compare differences between groups limited to two participant samples (Tabachnick & Fidell, 2013).

### 2.6. Results

There was a total of *N* = 135 participants in the current study, comprising *N* = 104 female, *N* = 26 male, *N* = 1 nonbinary, *N* = 1 nonbinary transfeminine, *N* = 1 trans male and *N* = 2 unspecified people aged 18–70 years (M = 34.45, SD = 16.30). All participants were UK residents. A priori power analysis was conducted using G*Power (Faul et al., 2009) to determine the required sample size for a binary logistic regression with six predictor variables. Assuming a medium effect size (Nagelkerke R^2^ = 0.13), an alpha level of 0.05 and a power (1 − β) of 0.80, the analysis indicated a minimum required sample size of 120–150 participants. The independent variables in the analysis were age, scores for the Coercive Control subsection of the MADVA Scale (Kirkman et al., 2025) and scores for the four factors of the PPTS-R (Boduszek et al., 2022). The dependent variables were the verdict decision (Guilty or Not Guilty) and scores from the JDS (Willmott et al., 2018).

Firstly, the relationship between age, the Coercive Control subsection of the MADVA Scale and the four factors of the PPTS-R—affective responsiveness (AFFRES), cognitive responsiveness (COGRES), interpersonal manipulation (INTMAN) and egocentricity (EGO)—were investigated using Pearson’s product–moment correlation coefficient. Preliminary analyses were performed to ensure there was no violation of the assumptions of normality, linearity and homoscedasticity. Visual inspection of histograms and Q-Q plots indicated that all continuous variables were normally distributed. Skew and Kurtosis values for all variables were within the threshold values of −1 and +1 and −2 and +2, respectively. This was supported by Shapiro–Wilk tests, which were non-significant for all variables (age, *W* = 0.80, *p* = 0.064; MADVA, *W* = 0.86, *p* = 0.097; AFFRES, *W* = 0.92, *p* = 0.075; COGRES, *W* = 0.98, *p* = 0.086; INTMAN, *W* = 0.97, *p* = 0.082; EGO, *W* = 0.97, *p* = 0.083), suggesting there was no significant deviation from normality. Scatterplots revealed linear relationships between the variables, and the spread of residuals appeared consistent across values, indicating that the assumption of homoscedasticity was met. No significant outliers were detected. Pearson correlation analysis indicated a number of statistically significant negative and positive correlations between several of the variables, as evidenced in Table 1.

### 2.7. Binary Logistic Regression

A Firth’s penalized binary logistic regression was conducted to examine the effect of a number of factors on the likelihood that respondents would report a Guilty or Not Guilty verdict. This statistical method was chosen to address the issue where less than 10% of the overall sample returned a Not Guilty verdict. The use of Firth’s method helps to effectively reduce bias and provide stable estimates despite a small sample size and rare event occurrence (Puhr et al., 2017; Wang, 2014). The model contained six independent variables (age, MADVA (CC) Scale scores and scores for the four factors of the PPTS-R). The full model containing all predictors was statistically significant (χ^2^ (6) = 13.81, *p* = 0.032), indicating that the model was able to distinguish between respondents who reported Guilty or Not Guilty verdicts. Only one of the independent variables made a statistically significant contribution to the model. The only predictor of returning a Not Guilty verdict was the MADVA (CC) Scale scores (β = 0.31, SE = 0.10, *p* = 0.001, OR = 1.36, 95% CI [1.12, 1.73]). The odds ratio indicated that respondents who had higher scores on the MADVA (CC) Scale were 1.36 times more likely to return a Not Guilty verdict than those who had lower scores on the MADVA Scale, controlling for all other factors in the model.

Three independent-samples t-tests were conducted to compare the scores on the Jury Decision Scale (JDS) for overall decision confidence and confidence in complainant and defendant believability between the participant groups returning Guilty and Not Guilty verdicts. Table 2 shows the overall Means and Standard Deviations for all tests. There was a significant difference in the overall decision confidence scores on the JDS between the two groups (t (133) = 3.16, *p* = 0.001, *d* = 1.15), meaning participants returning a Guilty verdict were more confident in their decision than participants returning a Not Guilty verdict. There was a significant difference in confidence in complainant believability scores between the two groups (t (133) = 4.92, *p* = 0.001, *d* = 1.79), meaning participants returning a Guilty verdict were more confident in their judgement of complainant believability than participants returning a Not Guilty verdict. There was a significant difference in the confidence in defendant believability scores between the two groups (t (133) = −2.90, *p* = 0.002, *d* = −1.06), meaning participants returning a Not Guilty verdict were more confident in the defendant believability than participants returning a Guilty verdict. The d values in all three tests indicated large effect sizes.

## 3. Discussion

This study aimed to assess whether juror characteristics, such as age, attitudes towards coercive control and psychopathic personality traits, can influence decision making in a mock coercive control trial and the subsequent verdict outcomes. The relationships between all independent variables were examined, and an analysis was run to investigate whether any of these independent variables could impact the likelihood of a Guilty or Not Guilty verdict being returned. Verdicts were further analysed using the JDS (Willmott et al., 2018) to assess whether there was a significant difference in scores for complainant and defendant believability and the subsequent verdict decisions. Additionally, overall confidence was assessed to see if there was a significant difference in confidence levels between Guilty and Not Guilty verdicts.

A significant positive correlation between MADVA (CC) Scale scores and all four subscales of the PPTS-R was found in the current study. Blair (1995) found that psychopathic individuals have reduced responsiveness to emotional stimuli and are unable to understand the discomfort of victims in comparison to non-psychopathic controls. When psychopathic individuals were given a violent scenario, for example, ‘a child pulling the hair of another child and the victim cries’ (Blair, 1995, p. 14), they were likely to be morally permissive and unlikely to consider the victim’s welfare. The disregard for victims and the inability to account for the damaging effect of violent behaviours seen in Blair’s (1995) study are arguably similar to the underpinnings of victim-blaming ideologies. Stark (2007) suggests victims of CC are blamed and judged by onlookers throughout the relationship as they generally stay with their abusive partner. Therefore, the link between psychopathic traits and accepting attitudes towards violence is unsurprising as impaired emotional responsiveness could potentially influence the endorsement of victim-blaming attitudes, as was supported by the findings of Watts et al. (2017).

The previous literature has found that both attitudes and psychopathic traits can influence decision making (Curley et al., 2022; Durand et al., 2017; Gunschera et al., 2022; Lilley et al., 2023; Stevens et al., 2023; Willmott et al., 2018, 2024). Therefore, it was assumed that both of these variables would be significant predictors of verdict outcomes. However, the current study only found MADVA (CC) Scale scores to be a significant predictor of returning Not Guilty verdicts. Attitudes can be developed through learning and experience (Pickens, 2005). Research has found that those involved in violent relationships, either as a victim or perpetrator, are more accepting of dating violence and tend to justify and minimise the severity of abusive behaviours (Dardis et al., 2016; Orpinas et al., 2012). This was also found in people in a relationship, irrespective of previous abuse experience (Erdem & Sahin, 2017). The relationship context influencing attitudes is crucial, as Strange et al. (2023) found that survey respondents from the general public were unable to recognise CC when it was displayed within an intimate relationship. However, altering the relationship context increased the likelihood of the same respondents identifying controlling behaviours (Strange et al., 2023). This highlights a certain level of expectation of violence within intimate relationships as these behaviours become normalised. Therefore, although the current study did not explore participants’ relationship status or experience of dating violence, the findings from Strange et al. (2023) provide further support for the suggestion that intimate relationship contexts can influence attitudes and perceptions of abuse. Therefore, regardless of the participants’ relationship status and their own experiences of violence, it is suggested that the intimate relationship context set out in the current study between the complainant and defendant was a significant factor influencing attitudes and the subsequent verdict decisions.

Furthermore, the current study found that PPTS-R scores were not a significant predictor of verdict outcomes, highlighting the dominant role of attitudes in underpinning decision making. Although there is a strong argument to suggest that PPT may influence decision making, this may only be apparent in certain contexts and when accompanied by negative victim attitudes, as previously suggested. While research on PPT and jurors is limited, the available research examining the influence of PPT has generally done so with regard to group deliberation, as opposed to solely investigating its influence on individual verdicts. Peace and Valois (2014) found that psychopathic traits are not substantial enough alone to influence individual decision making. Peace and Valois (2014) adopted a similar methodology to the current study, with a “he said, she said” ambiguous case of sexual assault, while involving variations in emotional display from the victim and perpetrator. Although traits did not significantly predict individual verdicts, the findings showed that higher scores for psychopathic traits influenced perceptions of storytelling, as those people were more likely to believe a victim was lying if they lacked emotional display (Peace & Valois, 2014). The findings of the current study were in line with those of Peace and Valois (2014) as psychopathic traits were correlated with more accepting attitudes surrounding CC. However, they were not the sole predictor of verdict outcomes. This is significant when considering juror characteristics and individual decision making as there is a clear relationship between psychopathic traits and attitudes. However, attitudes are the only significant predictor of subsequent verdict outcomes. Therefore, the findings of the current study add to the existing literature and provide foundational knowledge about the interplay between psychopathic traits, attitudes and individual decision making in the context of a mock coercive control trial.

Previous juror research has been criticised for using student samples (Nuñez et al., 2011), with Mills and Bohannon (1980) suggesting that decision making may be influenced by age. Therefore, the current study used a sample from the general public to ascertain whether age is significant in juror research. Attitudes were the only significant predictor of verdict outcomes; however, age was not correlated with attitudes, suggesting that acceptance of CC is not limited to either younger or older ages. Leisey et al. (2009) found that domestic abuse in long-term relationships continues into the victim’s old age. This suggests an ingrained pattern of abusive behaviours in perpetrators which may start at a young age and continue throughout the course of their life. This may also be the case for controlling behaviours; however, more research needs to be carried out to assess the maintenance of coercive control tactics in long-term relationships. Nevertheless, acceptance may derive from experience, suggesting there may not be variation in acceptance levels across generations. This may explain why age was not a significant predictor of verdict outcomes; it appears that experience, perceptions and attitudes are more important in juror decision making.

Complainant believability was associated with more Guilty verdicts and higher decision confidence, in line with previous research (Willmott et al., 2018). The confidence level is alarming as it suggests that those who return Not Guilty verdicts are less confident in their decisions yet the verdict has the same influence at trial. Considering the dominant role of attitudes throughout juror decision making, it is suggested that jurors undergo assessments to ascertain whether they bring undue bias to the trial to avoid uncertain verdict decisions based on problematic attitudes, stereotypes and misconceptions.

### 3.1. Limitations and Future Research

While the current study adds new and novel research findings to the literature, it is not without its limitations. Written vignettes have been praised for their usefulness when gaining information regarding attitudes and beliefs (Bieneck, 2015). This has evidently been successful in the current study, allowing the researchers to gain new information surrounding attitudes towards CC and their implications for a mock coercive control trial. However, the current study found an overwhelming majority of Guilty verdicts, which could be accounted for by considering the artificial nature of the vignettes, which may have swayed decisions as participants were aware they were not real court transcripts and were developed for the purposes of the study. Therefore, it could be argued that there was a social desirability bias, with participants choosing a Guilty verdict as they may have believed that it was the ‘right’ answer. Furthermore, as participants were aware that their decisions were only for the purposes of the study, they may have been more likely to choose a Guilty verdict as they were aware their decision lacked a real-world consequence (Bornstein, 2017; Diamond, 1997). Bornstein (2017) proposed the ‘consequentiality conundrum’, suggesting that, no matter how realistic a simulation is, it will always be just that, and lack real consequences. Miller and Bornstein (2013) argue that jurors’ decision making in real trials is affected by the “burden of justice”, referring to the responsibility given to jurors to make decisions that affect real people. This may explain why conviction rates for CC are limited while the current study shows a majority of Guilty and confident verdicts. It is suggested that real CC trials involve a lot of responsibility with a lack of physical evidence, which may result in lower conviction rates and less confidence. While the lack of consequence may have implications for trial simulation and juror research, it is unethical to deceive participants into believing their decisions do have consequences as it may cause psychological harm (Oates et al., 2021).

Further limitations pertain to the sample size obtained and the potential effects on the regression analysis undertaken. Firstly, it was gender skewed and future studies should aim to produce an even distribution of participants and adopt a non-opportunity sampling approach to address potential issues surrounding the generalisability of the findings. Secondly, although the regression model was statistically significant, indicating that it was able to distinguish between respondents who reported Guilty or Not Guilty verdicts, only eight respondents returned a Not Guilty verdict, as discussed above. This means that future studies wherein one of the binary groups is less than 10% of the obtained sample should incorporate a larger sample size to allow the regression analysis to be more sufficiently powered (Miles & Shevlin, 2001) to potentially confirm the current findings. Moving forward from this, future research should consider using an experimental design with an adapted real court transcript to aim to reduce social desirability and improve internal validity. This would also help to potentially account for the variability in decision consequence.

The current study aimed to examine individual juror decision making in a mock coercive control trial. Previous jury research has incorporated group deliberations within their methodology (Lilley et al., 2023; Sommers, 2006; Willmott et al., 2018); however, Curley et al. (2022) suggests that this can create additional bias in the decision-making process. Therefore, as the current study was assessing individual decisions, it was important not to include a group deliberation as this could have hampered the research aims. However, in light of the current research findings and considering the previous literature, it is suggested that the lack of group deliberation may have limited the potential significance of the role of PPT in decision making. The previous literature finding a significant contribution of PPT did so in the context of a deliberating jury (Lilley et al., 2023; Willmott et al., 2018), which may have mediated these outcomes. This could be significant, as research assessing individual decision making found that PPT had no effect on verdict decisions (Peace & Valois, 2014). Therefore, the findings of the current study add important relevance to the literature in that psychopathic traits may only be significant in the context of a deliberating jury. To further test this theory, future research could upscale the current study by incorporating a group deliberation element and using the JDS before and after deliberation to assess whether this has any influence on JDS scores and overall verdict decisions. Hedden (2017) suggests that group deliberation may include some members imposing pressure on other members of the jury, which may especially be the case for those who score highly for interpersonal manipulation. It could be suggested that those individuals could influence others’ JDS scores, altering believability from favouring the complainant to favouring the defendant. These prospective findings, alongside those of the current study assessing individual verdicts, could be crucial when considering the legitimacy of deliberation within real-life juries. The potential for PPT to only be significant in a deliberation context could raise important questions and concerns for the requirement of a deliberation at all.

### 3.2. Implications

The current research addresses gaps in the literature by measuring attitudes surrounding CC, as well as further analysing the implications of attitudes in a juror decision-making context. To the researchers’ knowledge, this is the first study of its kind. The findings from this study have important implications for future research as more work needs to be carried out to understand the processes of attitude formation with regard to coercive control specifically. The current study used the MADVA (CC) Scale, addressing male-perpetrated violence against female victims. It could be suggested within this context that acceptance of controlling behaviours is derived from sexism, misogyny and toxic masculinity, as Stark (2007) suggests that coercive control is mediated by male dominance and objectification of women. Therefore, the current study is important for understanding coercive control acceptance in the context of male-perpetrated violence against female victims. This is crucial as this encompasses the majority of domestic abuse cases (ONS, 2024b). As the findings suggest that attitudes are the only significant predictor of verdict outcomes, it could be suggested that attitudes are the main barrier to progressing through the CJS in the first instance. Research has shown that professionals are likely to dismiss victims’ accounts of psychological abuse (Murvartian et al., 2023), and the police are unable to recognise coercive control tactics (Barlow et al., 2019). This may be associated with the misogynistic underpinnings of coercive control attitudes in a male perpetrator–female victim context. Future research should aim to examine this association in more depth while accounting for potential differences in attitudes that are dependent on the gender identity of each partner. Depending on the outcomes of such research, specific training programmes may be suggested for police officers and other agencies within the CJS to tackle subconscious bias and improve case progression.

Not only do the findings of the current study impact the CJS and juries, but participation in the study may have positive implications for creating awareness of coercive control and could potentially influence help-seeking behaviours. The current study not only used a coercive control scenario, but also included a counterargument from the defendant that manipulates and justifies every behaviour. The scenarios included the subtle behaviours exhibited throughout CC; therefore, the current study creates awareness of what coercive control is, as well as the ways in which perpetrators aim to minimise their behaviours, therefore highlighting the complexity of CC. Participants from the current study may know someone experiencing this type of abuse and be more knowledgeable of the signs after participating in the study. Therefore, there are positive implications for awareness, support and potential help-seeking behaviours.

## 4. Conclusions

The main finding of the current study shows that attitudes surrounding coercive control are the most significant predictor of verdict outcomes in a mock coercive control trial, as opposed to age or psychopathic personality traits. Attitudes determined which ‘story’ participants believed, which influenced their verdict decision. It has been suggested that future research should aim to assess attitudes across different relationship contexts to gain a more in-depth understanding of the underpinnings of attitudes surrounding coercive control. To the researchers’ knowledge, there is currently no research exploring the influence of age, attitudes and psychopathic personality traits on individual verdict outcomes in a mock coercive control trial. Therefore, a major strength of the current study is its attempt to create a new and novel research area. The practical implications of this study were discussed, highlighting the importance of assessing attitudes prior to jury service as well as the general implications of raising awareness of the seriousness of CC in an attempt to increase help-seeking behaviours. Additional efforts need to be made regarding identification of CC tactics, and training programmes should be implemented within the police to increase the likelihood of these behaviours being noticed so that cases can progress throughout the CJS and to improve the likelihood of a jury being required in these cases.

## Figures and Tables

**Table 1:** Descriptive statistics and correlations for all continuous variables.

Variable	Age	MADVA (CC)	AFFRES	COGRES	INTMAN	EGO
Age	1					
MADVA	−0.12	1				
AFFRES	−0.20 *	0.35 **	1			
COGRES	0.06	0.20 *	0.52 *	1		
INTMAN	−0.27 **	0.21 *	0.62 **	0.21 *	1	
EGO	−0.24 **	0.28 **	0.71 **	0.48 ***	0.69 **	1
*M*	34.45	11.25	11.03	14.16	14.87	13.37
*SD*	15.96	3.44	3.40	3.35	5.08	3.73
*Min*	18	8	7	7	7	7
*Max*	70	22	20	25	29	21

Note: * *p* < 0.05; ** *p* < 0.01; *** *p* < 0.001. MADVA CC: Coercive Control subsection of the Modern Adolescent Dating Violence Attitudes Scale; AFFRES: affective responsiveness; COGRES: cognitive responsiveness; INTMAN: interpersonal manipulation; and EGO: egocentricity.

**Table 2:** Descriptive statistics, Means and Standard Deviations for verdicts and JDS scores.

JDS Scores	Participant Group	N	Mean	Std. Deviation
Overall Decision Confidence	Guilty	127	52.66	4.50
	Not Guilty	8	47.50	4.21
Complainant Believability	Guilty	127	27.38	3.99
	Not Guilty	8	20.25	3.81
Defendant Believability	Guilty	127	17.13	3.99
	Not Guilty	8	21.25	1.98

Note. JDS: Jury Decision Scale.

## Data Availability

The original contributions presented in this study are included in the article. Further inquiries can be directed to the corresponding author.

## Appendix A

### Appendix A.1. The Trial of Mark Williams

#### Appendix A.1.1. Background Information

Sally and Mark met on a dating website. They talked online for around a week before they met in person for a date. Mark brought Sally flowers, complimented her, and made her feel special. They instantly fell for each other and after a month of dating, they made it official, and Mark asked Sally to move in with him. This meant Sally had to move to a new city away from her family and friends. Sally was willing to make the move for Mark as she knew she’d be able to stay in touch with family and friends.

#### Appendix A.1.2. The Allegation

After a year of being in a relationship, Sally alleged that she was being coercively controlled by Mark.

#### Appendix A.1.3. Legislation

In England and Wales, Coercive Control is a criminal offence under Section 76 of the Serious Crime Act, 2015.

The offence of controlling or coercive behaviour is only applicable where:

- The victim and perpetrator are personally connected at the time the behaviour takes place.
- The behaviour has had a serious effect on the victim.
- The behaviour takes place repeatedly or continuously.
- The perpetrator must have known that their behaviour would have a serious effect on the victim.

The ways it can be proved the perpetrators behaviour has a ‘serious effect’ on the victim is:

- If it causes the victim to fear, or more occasions, that violence will be used against them; or
- If it causes the victim serious alarm or distress which has a substantial adverse effect on their usual day-to-day activities.

#### Appendix A.1.4. Juror Instructions

You are now a member of the jury that will require you to decide the guilt of the defendant, Mark Williams, who has entered a plea of Not Guilty to the charge of Coercive Control. Shortly, you will be asked to consider the evidence in this case before deciding whether you find him guilty or not guilty in relation to this charge. You are likely to see competing accounts of the same events and it is for you to determine what you consider factual. After you have read both accounts of what happened, you will be asked to return a verdict.

#### Appendix A.1.5. The Prosecution Case

##### Witness Testimony: Sally Davis—Complainant

I moved in with Mark. Mark works from home, so we could spend lots of time together. He earned enough money to support both of us, so he told me I didn’t need to work. Mark gave me £50 a week. I felt as if I owed him, so always made sure the house was spotless, and I earned my keep. Mark said this wasn’t necessary, but he always pointed out bits that I’d missed and would watch over me while I cleaned, so I had to make sure I did it right. I attempted social media influencing as I wasn’t working. Mark wanted my phone password and my social media passwords so he could check who I was talking to and block anyone he felt was trying to come onto me. After a few months, I decided I wanted to become a gym influencer and document my weight loss journey. When I was about to leave the house in my gym clothes, Mark started laughing at me and taking pictures. He does this a lot. He told me I looked ridiculous and that I had to go change. I always have to wear what Mark says. I changed into joggers and a hoodie, but Mark put his hand against the door so I couldn’t leave. I wasn’t allowed to ‘waste his money’ on a gym membership. I was about to eat dinner, when Mark took my food away as I shouldn’t be eating if I want to lose weight. He then decided I could only have one meal a day and he chose what I ate. He started making me do extreme home workouts. It was only then that I was allowed my one meal a day. I started to feel unwell. I was lethargic and feeling low mentally, so I booked a doctor’s appointment. Mark thought I was being pathetic, and attention seeking and made me cancel the appointment. He then took my phone away and only let me use it under his supervision. After around a year of being in a relationship with Mark, I could no longer leave the house. I tried to contact my family when Mark left the room as I was scared, but he’d deleted all my contact list. He also changed my social media passwords so I could no longer use it. I saw where he was keeping my phone, so once he’d fallen asleep, that’s when I rang the police.

#### Appendix A.1.6. The Defence Case

##### Witness Testimony: Mark Williams—Defendant

Sally moved in with me. I work from home so we could spend lots of time together. She didn’t need to work anymore because I could afford to look after us both and give her some of her own money. I gave her £50 a week, but she could have had more if she just asked. Sally was always cleaning and made sure the place was tidy. I liked to help by telling her when she’d missed a spot because I know she likes to be thorough. Sally wanted to be a social media influencer, but she would have had creeps in her messages, so I asked for her passwords to protect her and make sure no one bothered her. She didn’t seem to have a problem with this. Sally sometimes wears outfits that aren’t very flattering. I don’t mean to upset her, but I do ask her to change because I don’t want people to stare or make rude comments. I’m only looking out for her. I make sure she wears clothes that are appropriate so that people don’t judge her. She said she was starting a weight loss journey, that’s why I took pictures of her, just so she could see her progress. Gyms are useless for weight loss if you don’t diet as well. I started planning meals for Sally that would help her lose weight like she wanted. I encouraged her to do home workouts rather than spending her money at the gym. I figured she could save the money to buy something nice for herself. I don’t think Sally realised how much hard work it takes to lose weight. She started feeling lethargic because her body wasn’t used to dieting and workouts. It wasn’t a doctor’s concern. Doctors have more important things to worry about, so I told her to cancel the appointment. I didn’t want her using the internet to search for her symptoms as I knew she’d only worry, so I thought it would be best if she didn’t have her phone so she wouldn’t worry as much. I knew if I didn’t watch what she was doing on her phone, then she’d only make another doctor’s appointment, which is a waste of time. I had her phone most of the time for her own sake, but I didn’t delete her contact list, she must have done it by accident. Phones are always updating and malfunctioning. I also didn’t change her social media passwords; she must have been hacked. Next thing you know, I woke up and the police were arresting me.

## References

[B1-behavsci-15-00803] Barlow C., Johnson K., Walklate S., Humphreys L. (2019). Putting coercive control into practice: Problems and possibilities. The British Journal of Criminology.

[B2-behavsci-15-00803] Bate C., Boduszek D., Dhingra K., Bale C. (2014). Psychopathy, intelligence and emotional responding in a non-forensic sample: An experimental investigation. The Journal of Forensic Psychiatry & Psychology.

[B3-behavsci-15-00803] Bieneck S., Oswald M. E., Bieneck S., Hupfeld-Heinemann J. (2015). How adequate is the vignette technique as a research tool for psycho-legal research?. Social psychology of punishment of crime.

[B4-behavsci-15-00803] Blair R. (1995). A cognitive developmental approach to morality: Investigating the psychopath. Cognition.

[B5-behavsci-15-00803] Blais J., Forth A. E. (2013). Potential labeling effects: Influence of psychopathy diagnosis, defendant age, and defendant gender on mock jurors’ decisions. Psychology, Crime & Law.

[B7-behavsci-15-00803] Boduszek D., Debowska A., McDermott D., Willmott D., Sharratt K. (2022). Psychopathic personality traits scale–revised (ppts-r): Empirical investigation of construct validity and dimensionality in a forensic and non-forensic sample. Deviant Behavior.

[B6-behavsci-15-00803] Boduszek D., Debowska A., Willmott D. (2017). A new model of psychopathy. Custodial Review.

[B8-behavsci-15-00803] Bornstein B. H., Kovera M. B. (2017). Jury simulation research: Pros, cons, trends, and alternatives. The psychology of juries.

[B11-behavsci-15-00803] British Psychological Society (BPS) (2021a). Code of ethics and conduct.

[B10-behavsci-15-00803] British Psychological Society (BPS) (2021b). Code of human research ethics.

[B9-behavsci-15-00803] Buongiorno L., Mele F., Petroni G., Margari A., Carabellese F., Catanesi R., Mandarelli G. (2025). Cognitive biases in forensic psychiatry: A scoping review. International Journal of Law and Psychiatry.

[B12-behavsci-15-00803] Cox J., Edens J. F., Rulseh A., Clark J. W. (2016). Juror perceptions of the interpersonal-affective traits of psychopathy predict sentence severity in a white-collar criminal case. Psychology, Crime & Law.

[B13-behavsci-15-00803] Crown Prosecution Service (CPS) (2023a). Controlling or coercive behaviour in an intimate or family relationship|The crown prosecution service. *Cps.gov.uk*.

[B14-behavsci-15-00803] Crown Prosecution Service (CPS) (2023b). Expert evidence|The crown prosecution service. *Cps.gov.uk*.

[B15-behavsci-15-00803] Curley L. J., Munro J., Dror I. E. (2022). Cognitive and human factors in legal layperson decision making: Sources of bias in juror decision making. Medicine, Science and the Law.

[B16-behavsci-15-00803] Dardis C. M., Edwards K. M., Kelley E. L., Gidycz C. A. (2016). Perceptions of dating violence and associated correlates: A study of college young adults. Journal of Interpersonal Violence.

[B17-behavsci-15-00803] Day A., Bowen E. (2015). Offending competency and coercive control in intimate partner violence. Aggression and Violent Behavior.

[B18-behavsci-15-00803] Diamantopoulos A., Siguaw J. A. (2000). Introducing LISREL: A guide for the uninitiated.

[B19-behavsci-15-00803] Diamond S. S. (1997). Illuminations and shadows from jury simulations. Law and Human Behavior.

[B20-behavsci-15-00803] Durand G., Plata E. M., Arbone I.-S. (2017). Negative attitudes towards psychopaths: The role of one’s own psychopathic traits. Personality and Individual Differences.

[B21-behavsci-15-00803] Edens J. F., Guy L. S., Fernandez K. (2003). Psychopathic traits predict attitudes toward a juvenile capital murderer. Behavioral Sciences & the Law.

[B22-behavsci-15-00803] Ellison L., Munro V. E. (2009). Reacting to rape: Exploring mock jurors’ assessments of complainant credibility. British Journal of Criminology.

[B23-behavsci-15-00803] Ellsworth P. C., Hastie R. (1993). Some steps between attitudes and verdicts. Inside the juror: The psychology of juror decision making.

[B24-behavsci-15-00803] Enander V. (2010). Jekyll and hyde or ‘who is this guy?’—Battered women’s interpretations of their abusive partners as a mirror of opposite discourses. Women’s Studies International Forum.

[B25-behavsci-15-00803] Erdem A., Sahin R. (2017). Undergraduates’ attitudes toward dating violence: Its relationship with sexism and narcissism. International Journal of Higher Education.

[B26-behavsci-15-00803] Faul F., Erdfelder E., Buchner A., Lang A.-G. (2009). Statistical power analyses using G*Power 3.1: Tests for correlation and regression analyses. Behavior Research Methods.

[B27-behavsci-15-00803] Gillespie S. M., Lee J., Williams R., Jones A. (2022). Psychopathy and response inhibition: A meta-analysis of go/no-go and stop signal task performance. Neuroscience & Biobehavioral Reviews.

[B28-behavsci-15-00803] GOV.UK (2018). Your legal responsibilities as a juror.

[B29-behavsci-15-00803] Gunnell J. J., Ceci S. J. (2010). When emotionality trumps reason: A study of individual processing style and juror bias. Behavioral Sciences & the Law.

[B30-behavsci-15-00803] Gunschera L. J., Brazil I. A., Driessen J. M. A. (2022). Social economic decision-making and psychopathy: A systematic review and meta-analysis. Neuroscience & Biobehavioral Reviews.

[B31-behavsci-15-00803] Hare R. D. (1991). The hare psychopathy checklist-revised.

[B32-behavsci-15-00803] Hedden B. R. (2017). Should juries deliberate?. Social Epistemology.

[B33-behavsci-15-00803] Helm R. K., Growns B. (2022). Prevalence estimates as priors: Juror characteristics, perceived base rates, and verdicts in cases reliant on complainant and defendant testimony. Applied Cognitive Psychology.

[B34-behavsci-15-00803] Hritz A. C., Royer C. E., Helm R. K., Burd K. A., Ojeda K., Ceci S. J. (2015). Children’s suggestibility research: Things to know before interviewing a child. Anuario de Psicología Jurídica.

[B35-behavsci-15-00803] IBM Corp (2023). IBM SPSS statistics for windows *(Version 29.0.2.0)*.

[B36-behavsci-15-00803] Jisc (2025). Jisc online surveys.

[B37-behavsci-15-00803] Kirkman G., Willmott D., Boduszek D., Debowska A. (2025). Introduction and validation of the Modern Adolescent Dating Violence Attitude (MADVA) scale: A contemporary tool for assessing adolescent attitudes towards dating violence in offline and online environments. International Journal of Law, Crime and Justice.

[B38-behavsci-15-00803] Leisey M., Kupstas P. K., Cooper A. (2009). Domestic violence in the second half of life. Journal of Elder Abuse & Neglect.

[B39-behavsci-15-00803] Leith K. P., Baumeister R. F. (1998). Empathy, shame, guilt, and narratives of interpersonal conflicts: Guilt-prone people are better at perspective taking. Journal of Personality.

[B40-behavsci-15-00803] Lilley C., Willmott D., Mojtahedi D. (2023). Juror characteristics on trial: Investigating how psychopathic traits, rape attitudes, victimization experiences, and juror demographics influence decision-making in an intimate partner rape trial. Frontiers in Psychiatry.

[B41-behavsci-15-00803] Lonsway K. A., Fitzgerald L. F. (1994). Rape myths: In review. Psychology of Women Quarterly.

[B42-behavsci-15-00803] Menaker T. A., Cramer R. J. (2012). The victim as witness: Strategies for increasing credibility among rape victim-witnesses in court. Journal of Forensic Psychology Practice.

[B43-behavsci-15-00803] Miles J., Shevlin M. (2001). Applying regression and correlation: A guide for students and researchers.

[B44-behavsci-15-00803] Miller M. K., Bornstein B. H., Miller M. K., Bornstein B. H. (2013). The experience of jurors: Reducing stress and enhancing satisfaction. Stress, trauma, and wellbeing in the legal system.

[B45-behavsci-15-00803] Mills C. J., Bohannon W. E. (1980). Character structure and jury behavior: Conceptual and applied implications. Journal of Personality and Social Psychology.

[B46-behavsci-15-00803] Murvartian L., Saavedra-Macías F. J., Infanti J. J. (2023). Public stigma toward women victims of intimate partner violence: A systematic review. Aggression and Violent Behavior.

[B47-behavsci-15-00803] Nuñez N., McCrea S. M., Culhane S. E. (2011). Jury decision making research: Are researchers focusing on the mouse and not the elephant in the room?. Behavioral Sciences & the Law.

[B48-behavsci-15-00803] Oates J., Carpenter D., Fisher M., Goodson S., Hannah B., Kwiatkowski R., Prutton K., Reeves D., Wainwright T. (2021). BPS code of human research ethics. BPS Code of Human Research Ethics.

[B49-behavsci-15-00803] Office for National Statistics (ONS) (2024a). Domestic abuse in England and Wales overview: November 2024.

[B50-behavsci-15-00803] Office for National Statistics (ONS) (2024b). Domestic abuse prevalence and trends, England and Wales: Year ending march 2024.

[B51-behavsci-15-00803] Office for National Statistics (ONS) (2024c). Domestic abuse victim characteristics, England and Wales: Year ending march 2024.

[B52-behavsci-15-00803] Orpinas P., Hsieh H.-L., Song X., Holland K., Nahapetyan L. (2012). Trajectories of physical dating violence from middle to high school: Association with relationship quality and acceptability of aggression. Journal of Youth and Adolescence.

[B53-behavsci-15-00803] Patrick C. J., Fowles D. C., Krueger R. F. (2009). Triarchic conceptualization of psychopathy: Developmental origins of disinhibition, boldness, and meanness. Development and Psychopathology.

[B54-behavsci-15-00803] Peace K. A., Valois R. L. (2014). Trials and tribulations: Psychopathic traits, emotion, and decision-making in an ambiguous case of sexual assault. Psychology.

[B55-behavsci-15-00803] Pennington N., Hastie R. (1992). Explaining the evidence: Tests of the story model for juror decision making. Journal of Personality and Social Psychology.

[B56-behavsci-15-00803] Pickens J. (2005). Attitudes and perceptions. Organisational Behaviour in Health Care.

[B57-behavsci-15-00803] Pitman T. (2016). Living with coercive control: Trapped within a complex web of double standards, double binds and boundary violations. British Journal of Social Work.

[B58-behavsci-15-00803] Puhr R., Heinze G., Nold M., Lusa L., Geroldinger A. (2017). Firth’s logistic regression with rare events: Accurate effect estimates and predictions?. Statistics in Medicine.

[B60-behavsci-15-00803] Raine A., Uh S. (2018). The selfishness questionnaire: Egocentric, adaptive, and pathological forms of selfishness. Journal of Personality Assessment.

[B59-behavsci-15-00803] R Core Team (2025). R statistical software v4.5.0.

[B61-behavsci-15-00803] Serious Crime Act (2015). Legislation.gov.uk.

[B62-behavsci-15-00803] Singleton C. (2024). Understanding the jury selection process—Inside HMCTS. *Insidehmcts.blog.gov.uk*.

[B63-behavsci-15-00803] Sommers S. R. (2006). On racial diversity and group decision making: Identifying multiple effects of racial composition on jury deliberations. Journal of Personality and Social Psychology.

[B64-behavsci-15-00803] Stark E. (2007). Coercive control: How men entrap women in personal life.

[B65-behavsci-15-00803] Stark E., Lombard N., McMillan L. (2013). Coercive control. Violence against women: Current theory and practice in domestic abuse, sexual violence and exploitation.

[B66-behavsci-15-00803] Stevens K. L., Mojtahedi D., Austin A. (2023). Juror decision-making within domestic sex trafficking cases: Do pre-trial attitudes, gender, culture and right-wing authoritarianism predict believability assessments?. Journal of Criminal Psychology.

[B67-behavsci-15-00803] Strange C., Bartels L., Boxall H., Fitz-Gibbon K., Biddel N. (2023). Public attitudes towards coercive control: Evidence from a nationally representative population survey.

[B68-behavsci-15-00803] Tabachnick B. G., Fidell L. S. (2013). Using multivariate statistics.

[B69-behavsci-15-00803] Walker J., Kelty S. F., Tseung-Wong C. N. (2024). Bystander intervention in coercive control: Do relationship to the victim, bystander gender, and concerns influence willingness to intervene?. Journal of Interpersonal Violence.

[B70-behavsci-15-00803] Wang X. (2014). Firth logistic regression for rare variant association tests. Frontiers in Genetics.

[B71-behavsci-15-00803] Watts A. L., Bowes S. M., Latzman R. D., Lilienfeld S. O. (2017). Psychopathic traits predict harsh attitudes toward rape victims among undergraduates. Personality and Individual Differences.

[B72-behavsci-15-00803] Willmott D., Boduszek D., Debowska A., Woodfield R. (2018). Introduction and validation of the Juror Decision Scale (JDS): An empirical investigation of the story model. Journal of Criminal Justice.

[B73-behavsci-15-00803] Willmott D., Willmott D., Rafique A., Widanaralalage B. K., Agneswaran A. (2024). Investigating the role of psychopathic personality traits, gender and ethnicity in rape myth acceptance. Psychiatry, Psychology, and Law.

